# A Further Account of Fatty Degeneration of the Placenta, and the Influence of This Disease in Producing Death of the Fœtus, Hemorrhage, and Abortion

**Published:** 1853-07

**Authors:** Robert Barnes

**Affiliations:** M. D. (Lond.)


*A further account of Fatty Degeneration of the Placenta, and the influence of this Disease in producing Death of the Fœtus, Hemorrhage, and Abortion.* By Robert Barnes, M. D. (Lond.)—The author, after referring to a paper read before the Society, in 1851, thought that the histories of the cases now submitted would be found to throw much additional light upon the pathology of fatty degeneration in an organ in which the existence of this affection had only been recently established. These cases would also, in his opinion, lead confirmation to the view he had expressed of the importance of this affection as a cause of death of the fœtus, hemorrhage and abortion. In the first case, the fœtus had attained apparently the middle period of gestation. The mother had been conscious of the death of the fœtus; she was taken suddenly with slight pains, and the child and placenta all came away together. The placenta, like the child, was free from putrefaction when sent to the author. The maternal surface of the placenta was deeply divided by sulci. Its general aspect resembles that of the brain, both in color and subdivision. All the lobes had a pale, yellow, glistening appearance, like fat; pink or red in the sulci. In the sulci, between the lobes, there was evidence of the vascular connexion with the uterus. With these exceptions, the placenta had an ex-sanguine appearance. A section showed that the yellow, fatty appearance was much more marked on the decidual surface, while, on approaching the fœtal surface there was more appearance of blood. There was nothing like fresh, healthy placental tissue. Examined under the microscope, the villi taken from any part near the decidual surface were much altered; those from near the fœtal surface were less altered, but none were found perfectly healthy. The villi in the more consolidated lobes were brittle, misshapen; the vessels breaking up and losing their defined outline. The chorion was, for the most part, destroyed, and the nuclei in the walls of the vessels were enlarged and crammed with granules. In the less consolidated parts the vessels retained their proper size and shape. The author's view of the pathology of this case was, that the fœtus perished sometime before expulsion. All fœtal placental circulation had therefore ceased. The large vessels which still connected the placenta with the uterus, were the channels through which an imperfect utero-placental circulation was carried on up to the last. As there could have been no interchange between the maternal and fœtal blood, this circulation must have been simply to maintain a low degree of nutrition in the placenta. Granular degeneration had commenced prior to the death of the fœtus; but the granular conversion of the villi continued to increase after the death of the fœtus, until it had reached such an extent as to cut off so much of the remaining vascular communication as to leave attachment of the placenta to the uterus any longer impossible; and this gradual arrest of the circulation would explain the fact, that there was no hemorrhage at the time of labor. The next four cases were similar in general features to the last: abortion taking place from the fifth to the eighth month, the fœtus having died in utero, and the placenta presenting the characteristic conditions of the fatty degeneration just described. The sixth case was an abortion about the tenth week of gestation. There was some hemorrhage, and the ovum came away easily. The decidua in this case was very much thickened. On the uterine surface the oblique vascular openings were distinct, but it was pale and had a fatty appearance. Numerous granules of oil were seen in the cells of the decidua; the fœtal surface of the decidua presented similar appearance, but the fatty change was less extensive. The seventh case was also an abortion at the early period of pregnancy, and the examination of the parts of the ovum were given in detail. The author believed that these cases illustrated the influence of fatty degeneration in producing abortion, by rendering the villi of the chorion or placenta unfitted for their office of maintaining the nutrition of the embryo; and he thought the following considerations established the doctrine, that fatty degeneration of the placenta was really the cause of abortion: Firstly, there was no sign in case of decomposition of the placenta. Secondly, the presence of distinct indications of continued vascular connexion between the placenta and the uterus up to the moment of detachment and expulsion. Thirdly, the various degrees of progress of the degeneration in different portions of the same placenta. Fourthly, the obvious commensurate diminution of the vascular connexion between the placenta and uterus with the progress of the affection. Fifthly, the almost constant evidence of the death of the fœtus having taken place at some definite but not remote period. Sixthly, the analogical argument derived from the better known pathology of fatty degeneration in other organs; as in muscular structure of the heart, in the liver, in the kidneys, and in the bloodvessels, exhibiting unequivocally that this change took place during the life of the individual, and that, sooner or later, the resulting disorganization terminated in death. In conclusion, the author remarked, that fatty degeneration of the placenta was far from being an uncommon event. He exhorted practitioners to the employment of the microscope as the only means of investigating the abnormal conditions of the placenta. Alterations of structure in elementary tissues could not be recognised without the aid of that instrument, and he confidently believed that the morbid conditions described by various authors as examples of scirrhous disease, of atrophy, or of tubercle, might, by microscopical analysis, have been demonstrated as fatty degeneration The paper was illustrated by a number of carefully executed diagrams and microscopic drawings

Dr. Druitt could not let the opportunity pass of expressing his sense of the value of Dr. Barnes' researches in this new and hitherto unexplored department of pathology, from which many valuable results would probably be obtained hereafter. He remarked that it was important to collect all the facts possible, in order to discriminate trivial and accidental changes from those which were really important in the production of disease. He would mention, as an example, that state of villi of the chorion which was frequently found in abortion, in which they were bulged and ampullated, and apparently in an incipient state of cystic disease, which was believed to be the cause of the abortion. The villi of the non-placental portion of the chorion were commonly said to be absorbed after the earliest months of pregnancy; but he had constantly found them at the end of pregnancy, scattered over the chorion, of the size which they attain in the third month; but they were sometimes found in the secundines of the mature fœtus, distorted and enlarged, like those said to be in an incipient state of cystic disease, and usually loaded with dark granules of oil.

Dr. Barnes said that, considering the length of his paper, he was unwilling to trespass further upon the time of the society. He would limit himself to answering the question of Dr. Druitt. The fatty matter described as having been observed in the placental tissues had not been subjected to any test; it was a matter of simple observation. The granules or spherules of oil were characteristic in form and appearance, and it did not appear to him necessary to resort to any other test than direct observation, instructed by familiarity with the forms and appearances which fatty matter assumed in the placenta. The author was not prepared to admit that earthy or calcareous deposits had any essential or frequent connexion with fatty degeneration. He did not of course deny that the vessels of the placenta might be affected with a kind of earthy degeneration, like the arteries of the human body; but he repeated his belief that earthy deposits in the placenta did not necessarily, or in all cases, indicate a process simply the result of elimination from the blood, modified, probably by the conditions of pregnancy, and analogous, in this respect, to the effusion of fibrin he had adverted to in the paper. He wished to take the opportunity to enforce upon the attention of the Society, more clearly than the reading of the paper had permitted, the last case recorded. This was a case in which the child was born alive and healthy, at the full period, and in which, nevertheless, the placenta exhibited a considerable degree of fatty degeneration. There could not be a doubt therefore, that fatty degeneration might arise in the placenta, apart from the death of the fœtus, apart from mal-nutrition, or from any defect of development force. Had it been the fate of this child to have been retained a few weeks longer in the womb, it must inevitably have been cut off by the progress of the disease; but it so happened that the alteration began towards the termination of gestation, and had not made such advances as to destroy the function of the placenta before the natural period of delivery. This case supplied the last proof that was wanting to demonstrate one of the main propositions he had brought before the Society, and to prove the importance of this affection as a cause of death of the fœtus and abortion.—*Lancet.*—*N. O. Monthly Med. Register.*

